# An Old Practitioner, in Answer to Philo-Medicus

**Published:** 1802-03

**Authors:** 


### *To the Editors of the Medical and Phyfical Journal.*

GENTLEMEN,

I Am glad that the Philofophic world have not yet forbid the opinions of one, whofe years might probably bring upon him the appellation *doted.* Advanced age, and the various opportunities which I have had, both of feeing and knowing the world, encourage me to offer a few Remarks upon Philo-Medicus. Perhaps he may be fome Young Phyfician who wifhes well to the Character; but, I fear, he knows too little of the Character, and is fomewhat too faft.

If what follows be deemed worthy a place in your ufeful Journal, it will add to the happinefs of your conftant reader,

AN OLD PRACTITIONER.

---

Philo-Medicus feems to plume himfelf, that *his defign* has been happily accomplifhed, viz. the publication of an Annual Lift of Medical Practitioners. If it be *happily accomplifhed*, it is more than I can fee. It appears not only that fomething in the *plan* is wanting which is material, and for which I have reafon to believe he has not provided, but it is evident that the plan is very unhappily accomplifhed. "The only difficulty, fays he, that would occur, is in making out the firft lift; after that is accomplifhed, it would become a regular bufinefs," &c. Does he expect that the Medical Practitioners will be at the trouble and expence to fend letters containing their names, &c. poft paid, every year, becaufe it is *his plan?* If not, how will publifhers of Almanacs know when to blot out the names of thofe who have departed this life fince their names were inferted. He cannot expect that the relations of the deceafed will write to the publifhers for that purpofe, great part of them may never know of *his plan.*

Whether it be that I view my own end at no great diftance, and am thereby led to this objection, I will not fay; but it appears pears to me to be almoſt inſurmountable, as well as others I could point out.

Again, he ſays, "By theſe means every man might be informed of the regular practitioners in his part of the country; and if he afterwards employed an impoſtor, he had himſelf to blame." Now, from the above objection, it is evident the Almanac will be a very uncertain guide; and from the few who have thought it worthy their attention to ſend their names for inſertion at firſt, it cannot be truſted in future as a guide. Numbers of Medical Gentlemen laugh at the plan. What profit can a reſpectable practitioner promiſe himſelf, who has long practiſed in a town with reputation, by having his name in an Almanac? It is only adapted to thoſe whoſe names muſt thus be made public, otherwiſe their names would not be known, or thoſe who are young in buſineſs, and muſt be heard of.

But what can have ſet Philo-Medicus a railing againſt Phyſicians belonging to certain Colleges? If he looks around him, will he not ſee phyſicians belonging to every College who merit not the honourable name? He would do well to remember the old proverb, "Jack will never make a Gentleman," and change it a little, "A Degree will never make a Phyſician." How many phyſicians have I had occaſion to ſee, who, God knows, knew very little of their buſineſs, and would have looked upon a worthy practitioner as contemptible becauſe he was not a Fellow of the ſame College! It would be better for the world if ſterling merit were more prized than nominal honours. I glory to tell, that I have long practiſed under the banner of a Degree from the antient and honourable College of St. Andrews. The Profeſſors are well known to be Gentlemen of learning and probity, and wiſh to confer that honourary reward on thoſe only who deſerve it. They may be deceived. Other Colleges are alſo deceived. Is not an active, ſteady young man, who has ſerved an apprenticeſhip of five or ſix years, and who has ſeen a good practice during that time, well entitled to a degree, after being at college and practiſing for ſeveral years, although not ſo many years at college as is neceſſary to obtain an Edinburgh degree? Inſtead of this, nothing is now more common than to ſee a young man go all at once to College, attend it for three ſeaſons, pop into a druggiſt's ſhop occaſionally, or perhaps not at all, wait on certain gentlemen during his laſt ſeaſon, who tell him what to ſay and how to anſwer upon Examination, write his Diſſertation, and thus procure a degree for him from a moſt reſpectable College. Is not this deceiving the Profeſſors? And can the truth of this be denied? I have known young men made Doctors of Medicine by the moſt reſpectable College, in three years and nine months from from the time that they were entirely ignorant of Medicine and Languages. Never tell me of the ſuperlative honour of certain Degrees, until Profeſſors ſhall require of every Candidate to give oath that he did not attend theſe gentlemen called Grinders, and that the Diſſertation is his own production.

I could eaſily bring forward a number of reſpectable learned Phyſicians, who have their Degrees by means of what Philo-Medicus is pleaſed to call (being a ſweet ſounding name) a contemptible Certificate; and in contradiction to his affirmation, I have known Certificates given by Gentlemen of the firſt character, in order to obtain that honourary reward for thoſe who were well entitled to it by their ſuperior knowledge, and who could not from the time they were at College obtain it otherwiſe.

From what has been ſaid, however, I beg not to be underſtood as favouring any abuſe in the Study of Medicine, I would be the firſt to make it public; I only wiſh that practice were more attended to than ſpeculation, that ſenſe and worth were more eſteemed, and that young men would never be too haſty in their opinions.

*Scotland, Feb.* 7, 1802.

